# Metabolomic Responses of Guard Cells and Mesophyll Cells to Bicarbonate

**DOI:** 10.1371/journal.pone.0144206

**Published:** 2015-12-07

**Authors:** Biswapriya B. Misra, Evaldo de Armas, Zhaohui Tong, Sixue Chen

**Affiliations:** 1 Department of Biology, Genetics Institute, Plant Molecular and Cellular Biology Program, University of Florida, Gainesville, FL, 32610, United States of America; 2 Training Institute, Thermo Fisher Scientific, 1400 North point Parkway, Ste 10., West Palm Beach, FL, 33407, United States of America; 3 Department of Agricultural and Biological Engineering, University of Florida, PO Box 110570, Gainesville, FL 32611, United States of America; 4 Interdisciplinary Center for Biotechnology Research, University of Florida, Gainesville, FL 32610, United States of America; Universidade Federal de Vicosa, BRAZIL

## Abstract

Anthropogenic CO_2_ presently at 400 ppm is expected to reach 550 ppm in 2050, an increment expected to affect plant growth and productivity. Paired stomatal guard cells (GCs) are the gate-way for water, CO_2_, and pathogen, while mesophyll cells (MCs) represent the bulk cell-type of green leaves mainly for photosynthesis. We used the two different cell types, i.e., GCs and MCs from canola *(Brassica napus*) to profile metabolomic changes upon increased CO_2_ through supplementation with bicarbonate (HCO_3_^-^). Two metabolomics platforms enabled quantification of 268 metabolites in a time-course study to reveal short-term responses. The HCO_3_^-^ responsive metabolomes of the cell types differed in their responsiveness. The MCs demonstrated increased amino acids, phenylpropanoids, redox metabolites, auxins and cytokinins, all of which were decreased in GCs in response to HCO_3_^-^. In addition, the GCs showed differential increases of primary C-metabolites, N-metabolites (e.g., purines and amino acids), and defense-responsive pathways (e.g., alkaloids, phenolics, and flavonoids) as compared to the MCs, indicating differential C/N homeostasis in the cell-types. The metabolomics results provide insights into plant responses and crop productivity under future climatic changes where elevated CO_2_ conditions are to take center-stage.

## Introduction

Photosynthesis in green plants involves direct CO_2_ fixation by the action of ribulose-1,5-bisphosphate carboxylase/oxygenase (RuBisCO) in C3 species and phosphoenolpyruvate carboxylase (PEPCase) in C4 species. With the global climate change scenarios, a rising atmospheric CO_2_ concentration [1] can have a significant effect on plant physiology and metabolism and productivity [2]. A steady increase in atmospheric CO_2_ levels has been observed over the past 150 years, and this increase is projected to continue [3]. Although the effects of elevated CO_2_ on different plants have been studied, e.g., CO_2_ improves the photosynthesis of C3 plants, decreases stomatal conductance and photorespiration, and increases energy supply [4], our knowledge on the global metabolomic changes in different cell-types is extremely limited. While photosynthesis of C3 plants, including major crop species, is stimulated by an increase in the atmospheric CO_2_, photosynthetic capacity is often reduced after long-term exposure to elevated CO_2_, and there is no information available on short-term plant cellular responses to CO_2_ changes at the metabolomic scale. Under elevated CO_2_, an initial increase in the rate of carbon fixation was observed in many C3 plants, resulting in the accumulation of main products of photosynthetic carbon assimilation [5].

Omics tools enable systemic view of cellular physiology in a holistic manner to underscore the underlying metabolic networks and regulatory mechanisms. In addition, the use of single-cell types eliminates the ‘averaging effect’ of metabolomes that occurs in tissue or whole plant studies [6]. For a systemic understanding of cellular functions of GCs, metabolomics approaches have started to provide useful information [7]. Unfortunately, the currently known guard cell metabolome is small, with only about 105 metabolites and a majority of contribution from the reference plant *Arabidopsis thaliana* [8]. The putative roles of these metabolites in stomatal function are summarized in a recent review [9].

Elevated CO_2_ increases leaf area and numbers, branching, plant size and biomass, growth rates, C: N ratio and non-structural carbohydrates, in addition to reduced N-compounds such as amino acids and reduced allocation to phenolic compounds [10, 11]. In general, elevated CO_2_ may increase agricultural productivity (by increasing starch and sugars etc. by 10–20%) owing to enhanced photosynthesis and water use efficiency, mostly in C_3_ crops [12]. Transcriptomic studies conducted in several species such as *A*. *thaliana* [13, 14], rice (*Oryza sativa*) [15], and *Populus euramericana* [16] have allowed better understanding of the transcriptional basis of plant response to elevated CO_2._ Similarly, proteomic changes upon elevated CO_2_ were studied in the cash crop halophyte *Aster tripolium* L., which showed that elevated CO_2_ enabled an efficient ROS detoxification during salinity stress [17]. In light of the greenhouse effect, how the plants respond to elevated CO_2_ in their native growth environment has been the subject of many studies [18]. In *Rumex obtusifolius* L., 1000 ppm CO_2_ led to changes in the accumulation of most abundant metabolite oxalate in leaves as revealed by capillary electrophoresis mass spectrometry (CE-MS) analysis [19]. The CO_2_ environment plays a huge role in the metabolism of the algae [20]. An early work studied the effect of CO_2_ levels in *Chlamydomonas reinhardtii* cells, where 128 metabolites with significant differences between high- and low-CO_2_-grown cells were detected, and 82 were identified to include amino acids, lipids, and carbohydrates [21]. Among recent single-cell studies, *C*. *reinhardtii* proteome, transcriptome and metabolome have been studied under varying CO_2_ concentrations [22]. Furthermore, varying CO_2_ concentrations induced changes in 25% of the transcriptome in *C*. *reinhardtii*. Proteomic studies revealed the role of 22 extracellular proteins in *C*. *reinhardtii*, which are only expressed under low CO_2_ conditions [23]. Recently, using GC-ToF-MS, the CO_2_ responsiveness of 20 metabolites in GCs was analyzed [24]. How GCs sense the rising CO_2_ levels is intriguing as the cellular molecular basis of this response is far from being understood [25].

Atmospheric CO_2_ diffuses through the cell wall into the cytosol and dissolves in cell wall or apoplast water to form bicarbonate (HCO_3_^-^) [26]. The process of dissolving atmospheric CO_2_ in water, the subsequent processes of equilibration of dissolved CO_2_, HCO_3_^-^ and H_2_CO_3_, and the diffusion of those dissolved inorganic carbon to cells and the CO_2_ fixation sites in chloroplasts are slow physical and chemical processes [23]. An energized conversion of CO_2_ to HCO_3_^-^, however, occurs on the cytosolic side of the thylakoid membrane, i.e., hydration of CO_2_ to bicarbonate. Thus, we chose NaHCO_3_ treatment as a source of CO_2_/HCO_3_^-^ to investigate its effect on cellular metabolome. In addition, high HCO_3_^-^-mediated stomatal closure [27] and low HCO_3_^-^-induced stomatal opening [28] are well-known. HCO_3_^-^-induced stomatal movement involves H_2_O_2_ [29] and NO [27] mediated events. Study of the metabolomes of leaf cell-types over a time-course may help corroborating the earlier findings, and most importantly reveal novel metabolites involved in the HCO_3_^-^ response. This study on the effect of bicarbonate on the metabolomes of single-cell types in higher plants has not been reported.

## Materials and Methods

### Plant growth and authentic metabolite standards

Seeds of *B*. *napus* var. Global obtained from Svalöv Weibull AB (Svalöv, Sweden) were germinated in Metro-Mix 500 potting mixture (The Scotts Co., Marysville, OH, USA), and grown in growth chambers under a photosynthetic flux of 160 μmol photons m^-2^s^-1^ with a photoperiod of 10 h at 24°C in light and 20°C in dark. Fully expanded leaves from seven week-old plants were used for GCs and MCs enrichment experiments. The metabolite standards were obtained from Sigma-Aldrich (St. Louis, MO, USA). Stock solutions of the 330 compounds were dissolved in appropriate solvents, and prepared as a dilution series in water. The metabolite standards used for the HPLC-MRM-MS library can be found in S1 Table. These solutions were either used immediately or stored in -80°C. Serially diluted stock standard metabolite solutions, ranging in concentration of 0.1–100 pmol μL^−1^ were used to verify the linear response in the mass spectrometer.

### Isolation of GCs and MCs for HCO_3_^-^ treatment

Fifteen grams of leaves with main veins removed were blended four times for 15 s each in cold distilled water using a 14-speed Osterizer blender (Oster Inc., Boca Raton, FL). The blended mixture was washed with cold distilled water on a 100 μm Nylon mesh until the flow through was clear of MCs, debris, and plastids. These epidermal peels were then subjected to enzymatic digestion (0.7% Calbiochem cellulysin, 0.025% Macerozyme R10, 0.1% PVP 40, 0.25% BSA) for 50 min in a shaking water bath at 140 rpm in dark. The digest was collected on a nylon mesh (100 μm) and was repeatedly washed using 750 mL of Basic solution (560 mM sorbitol, 5 mM MES, 0.5 mM CaCl_2_, 0.5 mM MgCl_2_, 10 μM KH_2_PO_4_, pH 5.5) to remove broken epidermal (pavement) cells. The enriched GCs were then incubated in stomata opening buffer (50 μM CaCl_2_, 10 mM KCl, 10 mM MES-KOH, pH 6.2) prepared in Basic solution for an hour in the growth chamber under plant growth conditions. The GC preparations were freshly used.

MCs were isolated as previously described [30] except that the sucrose concentration was 0.7 M. The GCs and MCs were aliquoted in appropriate volumes, and NaHCO_3_ was added to a final concentration of 1 mM. Cells were incubated for 0, 5, 15, 30, 60, and 120 min on a shaker, and four replicates were generated for each data point. After treatment, the GCs and MCs were immediately frozen in liquid nitrogen and stored in—80°C until metabolite extraction.

### Stomatal aperture measurement

Stomatal apertures of the NaHCO_3_ treated GC preparations were measured using a Zeiss Axiostar Plus microscope (Carl Zeiss Microscopy, Thornwood, NY, USA). Sixty stomata were analyzed in each independent experiment and three such replicate observations were recorded. After incubation, 1 mM NaHCO_3_ was added to three independent samples where similar volume of H_2_O was used as control (mock). At different time points (0, 5, 15, 30, 60, and 120 min.) an aliquot was removed for observation. The results are presented as means ± SE. Data were analyzed using one-way ANOVA using DeviumWeb [31]. A P-value < 0.05 was considered as statistically significant.

### Targeted profiling of HCO_3_^-^-responsive metabolomes by HPLC-MRM MS

Targeted metabolite profiling of the untreated and HCO_3_^-^ treated GCs and MCs was performed using HPLC-MRM-MS/MS. Extraction of cellular metabolites was performed as previously described [32]. Extracted metabolites were dissolved in 100 μL H_2_O containing three internal standards (33 pmol lidocaine (positive mode), 210 pmol camphor-10-sulfonic acid and 100 pmol adonitol (ribitol) (negative modes) to compensate for retention time shift. The HPLC (Agilent, Santa Clara, CA, USA) used an autosampler (Agilent, Santa Clara, CA, USA) to inject the samples automatically. A C18-reverse phase (Gemini 5μ; 150 × 2.0 mm, Phenomenex, Torrance, CA, USA) analytical column was used with a C18 guard cartridge. The mobile phase consisted of two solvents, i.e., 0.1% formic acid in H_2_O (solvent A) and 0.1% formic acid in acetonitrile (solvent B). The HPLC method was run with the following gradient program, i.e., 1:99, v/v, at 0 min; 1:99, v/v, at 0.2 min; 99.5:0.5, v/v at 31 min; 99.5:0.5, v/v at 34 min; 1:99, v/v, at 34.2 min; 1:99 at 60 min at room temperature.

The HPLC was coupled to a hybrid triple quadrupole-ion trap (4000 Q-TRAP, AB Sciex, Foster City, CA, USA) mass spectrometer equipped with a TurboIonSpray (TIS) interface operated in both positive and negative ion modes. The electrospray ionization (ESI) parameters were: curtain gas (CUR) at 30 psi, the ion source (IS) at (+/-) 4500 V, nebulizer gas (GS1) at 50 psi, TIS gas (GS2) at 55 psi and the TIS probe temperature at 350°C. To optimize analyte sensitivity, individual working solutions having the characteristic MRM transition with the optimal declustering potential (DP), collision energy (CE), cell exit potential (CXP) were selected for individual metabolites. Each transition was performed with a 15–40 ms dwell time to get a scan time around 1.2 ms for all the transitions analysed using a method including an enhanced MS scan and an enhanced product ion scan before switching to MRM mode. Essentially, QQQ experiments scans [33] were acquired as MRM experiments where a specific set of MRM transitions were monitored for each run period according to the metabolites eluted within this particular time-frame. We monitored 59 (5.8 min), 40 (9.9 min), 45 (7.9 min), 31 (12.5 min), and 18 (23.8 min) MRM transitions over five periods for the positive ionization mode. For the negative ionization mode, 66 and 11 MRM transitions were recorded for the first and second periods, respectively. Thus, each MRM transition was obtained with a 5-ms dwell time, and the scan mode change was set for 700 ms. The total cycle time was 1–1.5 s for the period, which ensured at least 10 points were obtained for each eluted peak. Data were processed using Analyst^TM^ software version 1.5.1 (AB Sciex, Foster City, CA, USA) while peak areas were integrated using the IntelliQuan algorithm of the MuliQuant^TM^ software version 2.1 (AB Sciex, Foster City, CA, USA). The optimized parameters used for MRM experiments for each standard metabolite included in HPLC-MRM-MS/MS library are provided (S1 Table). Multiple blank solutions were prepared by adding only extraction solvent (buffer) and distilled water, respectively, whereas pooled samples were used as quality control (QC) runs for monitoring the chromatography and mass spectrometer conditions.

### Profiling of HCO_3_^-^-responsive metabolomes by GC-MS

Gas chromatography-mass spectrometry was performed as described [34]. Metabolite extracts were dried under vacuum without heating, and were then sequentially derivatized with methoxyamine hydrochloride (MeOX) and *N*-methyl-*N*-trimethylsilyl-trifluoroacetamide (MSTFA) [34]. Derivatization was carried out by adding 50 μL of MeOX (20 mg mL^-1^) in pyridine and shaking at 30°C for 90 min. followed by trimethylsilylation for 30 min at 37°C by addition of 100 μL MSTFA. One microliter of the derivatized sample was injected in splitless mode into a GC-MS system consisting of an autosampler and a TSQ8000 Triple Quadrupole (Thermo Scientific Inc., San Jose, CA, USA) or a 5977A Series GC/MSD (Agilent Technologies, Santa Clara, CA, USA) equipped with an electron impact (EI) ionization source. Injection of the sample was performed at 250°C with helium as a carrier gas and flow set to 2 mL min^-1^. GC was performed using a TR-5MS or HP-5MS capillary column (30 m × 0.25 mm × 0.25 μm). The temperature program started isothermal at 70°C for 1 min followed by a 6°C min^-1^ ramp to 300°C and a final 11 min hold at 300°C. The system was then temperature-equilibrated at 70°C for 5 min before the next injection. Mass spectra were collected at 20 scans/s with a range of *m/z* 40–600. The transfer line and the ion source temperatures were set to 250°C. A standard alkane mixture (C_8_-C_40_) was injected at the beginning and end of the analysis for tentative identification and monitoring shifts in retention indices (RI).

### Processing of raw mass spectrometry data

#### GC-MS data analysis

The GC-MS data were aligned and processed as described [35]. Aligned data were deconvoluted using Automated Mass Spectral Deconvolution and Identification System (AMDIS, National Institute of Standards and Technology, USA). The filtered raw GC-MS data comprised of data from four biological replicates and 73 curated analytes. Peak areas of the mass fragments (*m/z*) were normalized against the internal standard (adonitol). Metabolites were identified by comparing fragmentation patterns available in the MSRI spectral libraries of Golm Metabolome Database available from Max-Planck-Institute for Plant Physiology, Golm, Germany (http://csbdb.mpimp-golm.mpg.de/csbdb/gmd/gmd.html) by matching the mass spectra and RI [36]. Additional peak identification was confirmed by comparing against the NIST Mass Spectral Reference Library (NIST08/2008, National Institute of Standards and Technology, USA). Peak finding and quantification of selective ion traces were accomplished using AMDIS software. As a rule, if a compound had an AMDIS match factor of >60% and a probability score >20% as well as a matching RI to a known compound it was considered “probable”. Peak areas were normalized by dividing each peak area value by the area of the internal standard for a specific sample, and were further median normalized. Individual metabolite quantification data were assembled into a joint data set which included those measured by the targeted HPLC-MRM-MS.

#### Statistical analysis

Statistical analyses were performed using the statistical software R (Version 2.9.1, R Development Core Team 2007, http://www.R-project.org) [37]. When data were not normally distributed, normal transformations of continuous variables were attempted using DeviumWeb [31]. Normalized, transformed, imputed, outlier removed, and scaled peak area representative of relative metabolite amounts are presented in tables and figures. Values reported in all tables and text are presented as means, and differences were always considered significant when P < 0.05. **Univariate analysis:** Creation of the heat map and Student’s *t*-test (ANOVA-background) were performed using Microsoft Excel (Microsoft Corporation, Seattle, WA, USA). For heat maps, the data were normalized using z-scores of the intensity counts for each of the metabolites under the peak areas. The *t-*test was performed two-sided with equal or unequal variance, where the P-values were adjusted by Benjamini-Hochberg correction (BH) [38]. ANOVA was used to test for differences of the final model’s selected variables between cell-types with HCO_3_^-^-and control (H_2_O) treatment. The probability level for the test statistics was set at α = 0.05 and was adjusted for multiple hypotheses testing using BH to allow for a maximum 5% probability (q = 0.05) in false positive detection. Hierarchical clustering analysis (HCA) using average linkage clustering was performed on Euclidean distances using PermutMatrix [39]. Volcano plots were constructed using Multiplot version 2 available at GenePattern server [40]. **Multivariate analysis:** Principal components analysis (PCA) was performed at DeviumWeb [31], where output consisted of score plots to visualize the contrast between different samples and loading plots to explain the cluster separation. The data file was scaled with unit variance without any transformation. O-PLS-DA was used to highlight differences between control and HCO_3_^-^-treated metabolic phenotypes, for time-points and cell-types used in the study. Orthogonal partial least-squares discriminant analysis (O-PLS-DA) model was developed and plotted to explore the variation in metabolites, visualize their discrimination power, and determine their importance in predicting cellular response to treatments and time-points.

### Pathway enrichment and clustering analysis

Pathway enrichment was performed at Metaboanalyst (www.Metaboanalyst.ca) [41]. Briefly, increased or decreased metabolites that changed by 1.2 folds (increase) and 0.8 fold (decrease) were used as cutoffs across the biological replicates, treatments, and time-points indicating that the specific pathway enrichment levels would not be expected by random chance. In addition, pathway mapping were done using MBRole (http://csbg.cnb.csic.es/mbrole/), MSEA (http://www.msea.ca/MSEA/) and MetPA (http://metpa.metabolomics.ca/MetPA/) were used for additional over-representation analyses. For ID conversions, the Chemical Translation Service (CTS: http://cts.fiehnlab.ucdavis.edu/conversion/batch) was used to convert the common chemical names into their KEGG, HMDB, and InChiKeys (S1 Table). For clustering of time-course patterns, Short Time series Expression Miner (STEM) was used as a Java implementation with a graphical user interface (http://www.cs.cmu.edu/~jernst/st/) [42].

## Results

### HCO_3_^-^-mediated stomatal movement in *B*. *napus*

**Fig 1:**
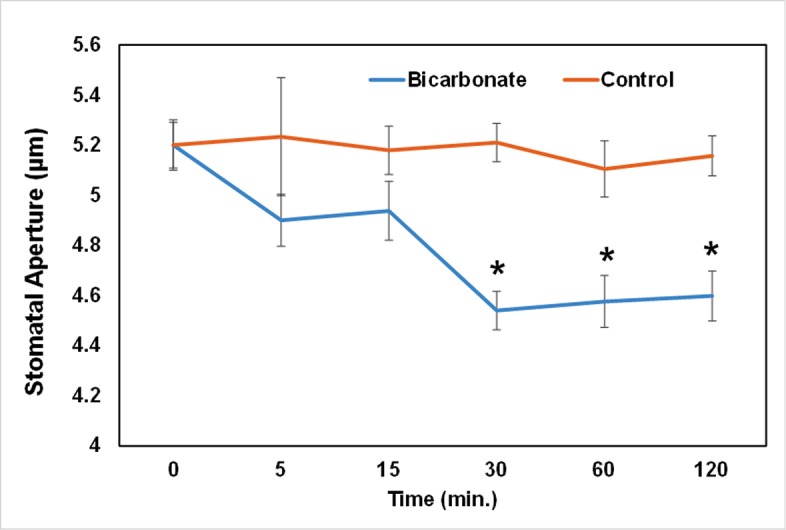
Stomatal movement in response to 1 mM HCO_3_^-^ added at time 0 min. Data were obtained from 60 stomata in three independent experiments and presented as means ± SE. The asterisks indicate significantly different mean values at P <0.05.

To test the effect of HCO_3_^-^ on *B*. *napus* stomatal movement, we administered NaHCO_3_ at concentrations of 0 (control) and 1 mM (treatment) to the isolated stomatal GCs (Fig 1). The results clearly showed that 1 mM HCO_3_^-^ was effective in significant reduction of stomatal aperture beyond 30 min post incubation (mpi) as compared to the control. In comparison, MCs did not show any recordable phenotypic changes under 1 mM HCO_3_^-^ treatment.

### Cataloging the MC and GC metabolomes and their responses to HCO_3_^-^ treatment

Using a two-pronged approach of targeted HPLC-MRM-MS and GC-MS, we detected and quantified a total of 268 metabolites in MCs and GCs at various time-points across four biological replicates (S1 and S2 Tables). These metabolites covered various metabolic pathways such as purine, pyrimidine, amino acid, central carbon, TCA, sugar, glyoxylate, phenylpropanoid, and flavonoid metabolism that were spread over the KEGG metabolic map (S1 Fig). The root mean square deviation (RSD) analyses of the data indicated that about 95% showed 10–20% RSD for each of the cell types, while the rest 5% were distributed between 30–40%.

The HCO_3_^-^ treated MC and GC metabolomes showed quantitative variations during the time-course experiment. The shared metabolites in MCs and GCs showed differential levels of changes (fold change cut-offs of < 0.8 and >1.2, at P < 0.05) (S3 and S4 Tables). To understand the detailed information arising from the multivariate analyses and to identify metabolite features that are significantly different between each time point of HCO_3_^-^ treatment, univariate statistical analyses including t-tests, fold change (FC) and volcano plots were conducted. The Volcano plots revealed that throughout the entire time-course, mostly the amino acids showed significant increases in MCs (except for 30 mpi), with increased accumulation of phenylpropanoids at 30 mpi and 60 mpi only (S2 Fig). In contrast, in the early time-points of 5, 15 and 30 mpi sugars, sugar phosphates, and organic acids showed significant increases in GCs, while decreases of phenylpropanoids and flavonoids were noted (S3 Fig). In addition, reduced levels of some phytohormones at 60 mpi and increases in some flavonoids and phenylpropanoids at 120 mpi were also noted in the GCs.

Between subject-ANOVA analysis using the three factors as cell-type (MC vs. GC), treatment (control vs. HCO_3_^-^), and time-course (0–120 mpi) indicated that HCO_3_^-^ treatment showed tremendous effect on purine, pyrimidine, flavone and flavonol, and alkaloid metabolism whereas time-effect was prominent in pyrimidine, arginine, and glyoxylate metabolism in GCs (S5 Table). In contrast, the MCs showed significant enrichment of taurine, valine, glutathione, amino acid, glycine, serine and nitrogen metabolism after the HCO_3_^-^ treatment, whereas time-effect showed enrichment of amino acid, fructose, and aminosugar metabolism (S5 Table). Although in GCs time and treatment showed interaction for metabolites enriched in pyrimidine, arginine, proline, and glyoxylate metabolism, such interactions were missing in MCs.

### Temporal metabolomic changes in HCO_3_^-^ treated MCs and GCs

**Fig 2:**
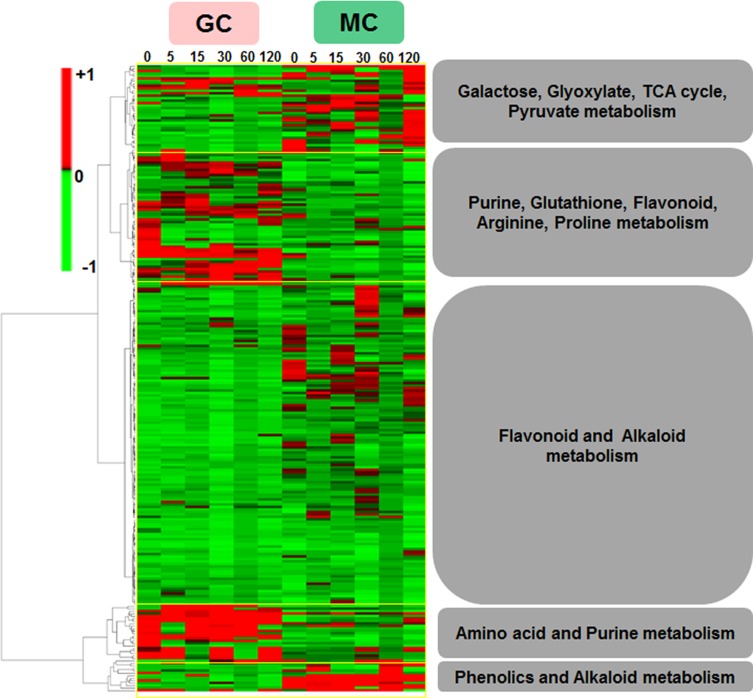
Hierarchical cluster analysis (HCA) of mean values of metabolite contents from four biological replicates showing 268 metabolites common to the two cell-types depicting the data structure dependent on the cell-types and time course (0–120 mpi) of HCO_3_^-^ treatment. Red and green indicate high and low concentrations of metabolites, respectively. Values were subjected to average linkage clustering (Euclidean distance). Outlined blocks in yellow show grouped metabolites in the two cell-types.

Many metabolites are shared between MCs and GCs at different time points. Six and 13 metabolites (enriched in phenylalanine and vitamin B6 metabolism) were common to all the five time-points for MCs and GCs, respectively (S6 Table). In addition, hydroxyflavone, vanillin and hexadecanoic acid were common at 5 mpi, 60 mpi and 120 mpi, respectively. Other significantly changed metabolites common between MCs and GCs include 1-ACC, 6-furfurylaminopurine, allantoin, creatine, GSH (reduced glutathione), L-homomethionine, indole butyric acid, and lactose. There were 52 metabolites common to the two cell-types that changed significantly at least in one cell-type at one time point (S7 Table). Hierarchical clustering analysis (HCA) was performed to classify metabolites and time-course profiles into clusters of different trends of changes (Fig 2). Interestingly, we found that these time-resolved metabotypes were discriminated into five distinct clusters. In the first cluster, galactose, glyoxylate, TCA cycle, and pyruvate metabolites were grouped, where increases were mostly in the MCs. In the second cluster purine, arginine, proline, glutathione, and flavonoid metabolites showed large increases in GCs compared to the MCs. The largest and third cluster grouped metabolites of mostly secondary metabolism origin and belonged to flavonoid and alkaloid biosynthesis, which showed increases in MCs and sharp decreases in GCs. The last two small clusters contained amino acid and purine metabolites with increases in GCs, and phenylpropanoid and alkaloid metabolites with increases in MCs.

**Fig 3:**
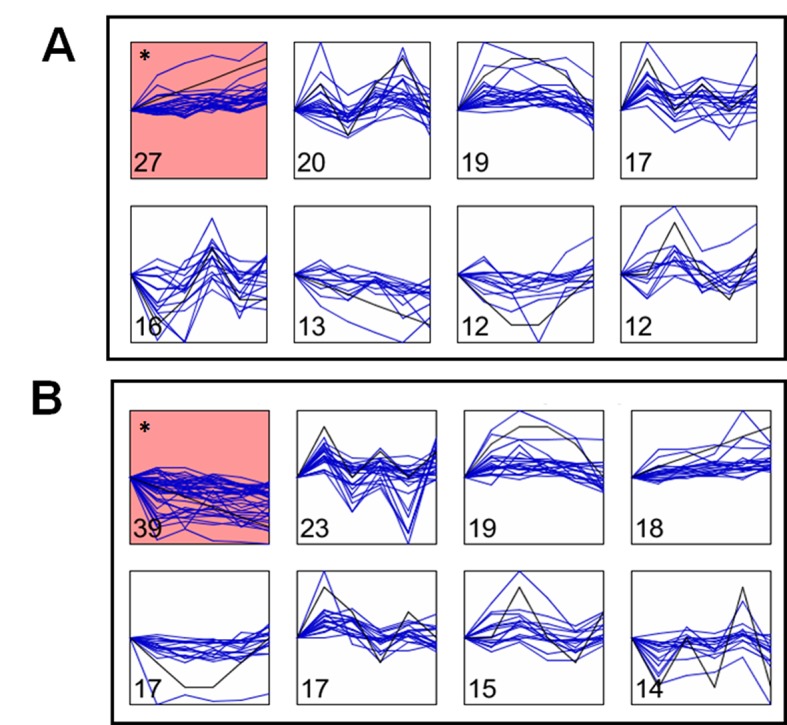
STEM analysis showing metabolite accumulation patterns across the time-course of HCO_3_^-^ treatment of (A) MC and (B) GC. Numbers at the bottom-left indicate the metabolites with similar trends, and the star indicates significance (P < 0.05).

In the short time-series expression miner (STEM) analysis, a model significantly explained the constantly increasing accumulation pattern of 27 metabolites in MCs (enriched in galactose, flavonoid, and pyruvate metabolism) (Fig 3A), whereas in GCs another model significantly explained the constantly decreasing pattern of 39 metabolites (enriched in pyrimidine, alanine, beta-alanine, aspartate, glutamate, pantothenate, and nitrogen metabolism) (Fig 3B) during the time-course. While a few of the models demonstrated clear and linear patterns of changes, and a majority of the metabolites showed mostly ‘biphasic’ patterns with two peaks and two troughs.

### Global and pathway-wise metabolic responses to HCO_3_^-^ treatment in GCs and MCs

**Fig 4:**
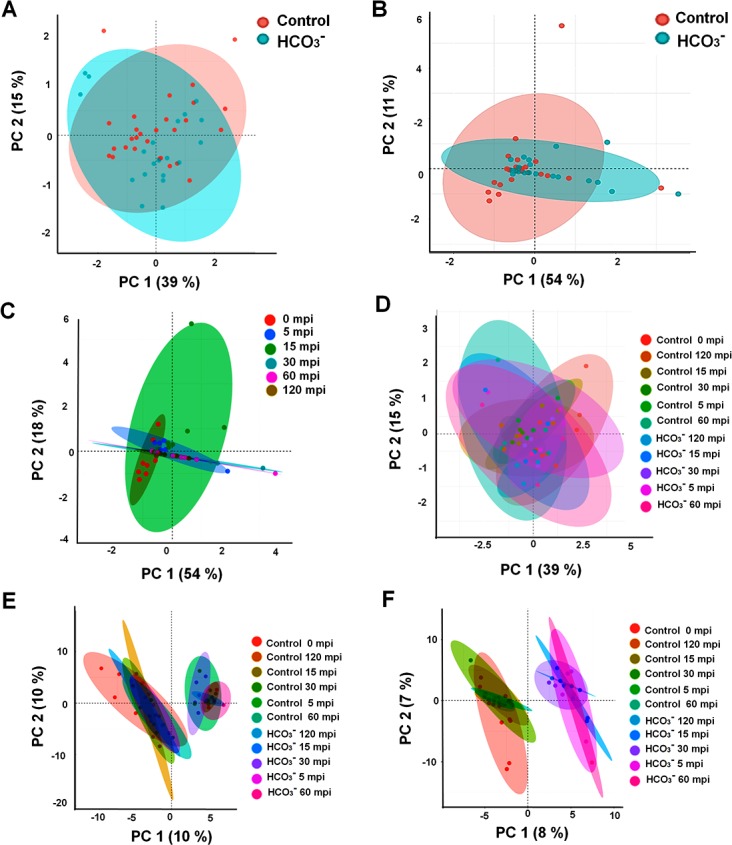
Principal component analysis (PCA) and orthogonal partial least square discriminant (OPLS-DA) analysis of metabolite changes in GCs and MCs after HCO_3_^-^ treatment. PCA was performed using four replicate data of relative metabolite abundances in the cell-types at 0, 5, 15, 30, 60, 120 mpi, and the generated PC1 and PC2 were plotted. PCA of two cell-types showing a clear separation of the two groups based on the 268 metabolites for the effect of treatments in (**A**) GCs, and (**B**) MCs. The effects of only **(C)** time and both (**D**) ‘treatment x time’ were displayed. In OPLS-DA, the metabolite changes as a result of interactions among ‘cell-type x treatment x time were displayed in **(E)** GCs and (**F**) MCs.

PCA using an unsupervised multivariate linear model revealed grouped and differential responses of the cell-types to HCO_3_^-^ with an interpretable visualization. The resulting plots for MCs explained 54% variations by two PCs (PC1- 39% and PC2-15%) attributed to the variations from the HCO_3_^-^ treatment and time course components (Fig 4A). On the other hand, the resulting plots for GCs explained 72% variations by two PCs (PC1-54% and PC2-18%) attributed to the variations from the HCO_3_^-^ treatment and time course components (Fig 4B). The effect of time (Fig 4C) and treatment x time (Fig 4D) were also discernible using the global PCA. A supervised approach of classification, OPLS-DA analysis was conducted to indicate grouped responses of the cell-types to treatment and time-course for both MCs and GCs. The grouped responses of cell-type x treatment x time are shown for the two cell-types (Fig 4E and 4F).

**Fig 5:**
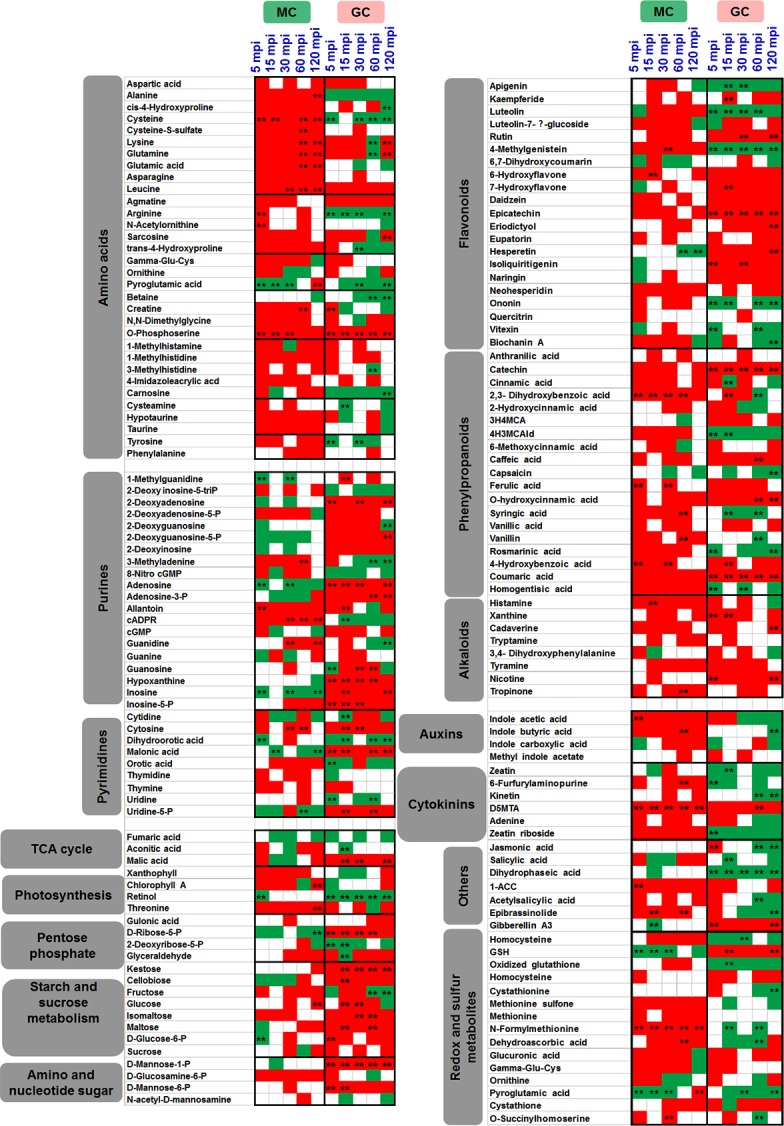
Responses of major metabolite groups in MCs and GCs upon HCO_3_^-^ treatment in the time-course study. Green and red squares indicate decreased (<0.8) and increased (>1.2) fold changes, respectively. The asterisks indicate significant changes (P <0.05).

**Fig 6:**
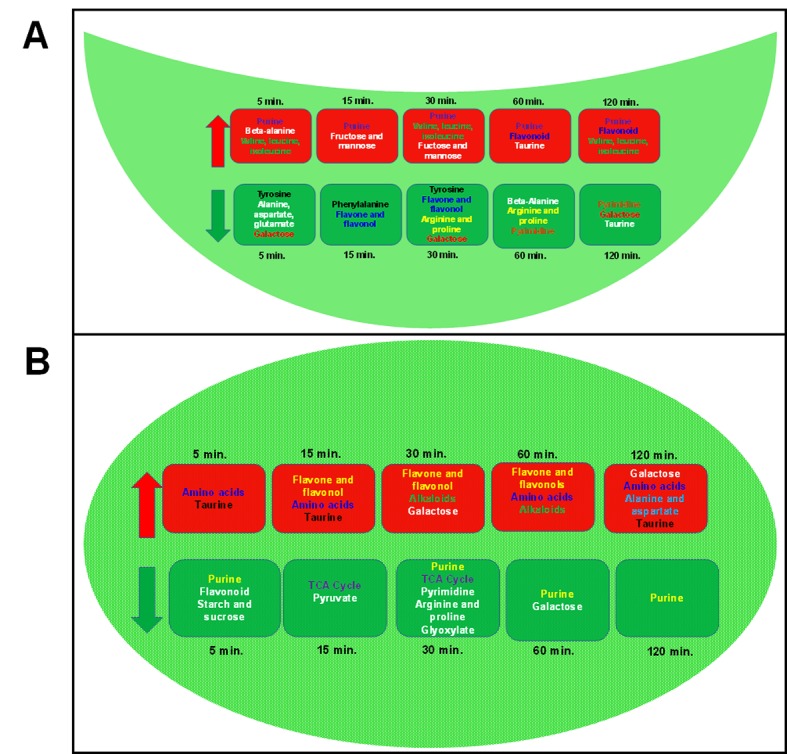
Summary of the increased and decreased metabolic pathways in (A) GCs and (B) MCs after HCO_3_^-^ treatment.

In our metabolomic analyses, 26 and 27 metabolites in MCs and GCs were uniquely identified, respectively, to be enriched with sugar alcohols, phytohormones, organic acids, and aromatic amino acid metabolism in MCs and sugar alcohols, flavonoids, organic acids, sugars, and fatty acid in GCs (S4 Fig). Out of the unique metabolites, glycine, proline, and ABA showed significant increases at 5 mpi in MCs, while ethylene glycol, myricetin, niacin, suberylglycine, succinic semialdehyde, and uric acid showed significant decreases at 60 mpi in GCs (S4 Fig). In addition, the number of increased metabolites outnumbered the decreased in the two cell-types (S5 Fig), which corroborated the recent transcriptomic findings [43]. Furthermore, there more number of significantly changed metabolites in GCs than in MCs (S2 Fig). Hence, we looked at the major metabolic pathways in both cell-types to gauge the impact of treatment. Changes in amino acids demonstrate a very clear increased accumulation in MCs as compared to decreases in GCs (Fig 5). This preference of MCs for C-incorporation into N metabolism is opposite to GC response in decreased N-accumulation. Significant increases and decreases in cysteine levels were observed in MCs and GCs, respectively. Pyroglutamate and o-phosphoserine showed opposite trends in the cell-types, i.e., significant decreases in MCs and increases in GCs. In terms of primary metabolism, which centers on the central C-metabolism, the TCA cycle remained unchanged attributable to high fluxes in the cycle (Fig 5). However, in GCs, the continual and significant increase of malate at all time-points of HCO_3_^-^ treatment indicates induction of glyoxylate cycle thus leading to gluconeogenesis. These elevated glyoxysomal responses are further supported by continually elevated succinic acid in the GCs (S5 Fig), which is released during acetyl CoA utilization and used in carbohydrate biosynthesis. However, the photosynthesis-related metabolites showed increasing trends in MCs and opposite trends in GCs. In contrast, the GCs showed higher accumulation of pentose phosphate pathway metabolites than MCs. Following the same patterns, starch and sucrose metabolites, and amino and nucleotide sugar metabolites in GCs showed significantly higher accumulation than in MCs. In GCs, fructose, glucose, and mannose showed increases during the early time-points, while kestose, maltose, and isomaltose showed sustained increases throughout the time course (Fig 5). In contrast, changes of the above metabolites in MCs were not significant. Another important and large pool of cellular N-metabolites contained purines and pyrimidines. In GCs, increases in purine metabolism are clearly visible during the entire time-course, whereas pyrimidine metabolism showed mixed responses across the time-points. On the other hand, in MCs purine metabolites were constantly decreased throughout the time-course, except for 15 mpi (Fig 5; S7 Table). In addition, the pentose phosphate pathway metabolites that are interconnected to purine and pyrimidine metabolism, i.e., glucose-6-phosphate and ribose-5-phosphate levels showed increased accumulation in GCs throughout the time-course, indicating the need for cellular energy (NADPH and ATP) for stomatal movement. On the other hand, this response was opposite in MCs where these two metabolites were decreased. Over all, the changes in purine and pyrimidine metabolites showed clear increases in GCs (Fig 6). Secondary metabolites play important roles in plant defense responses and are derived from primary C-and N-metabolism. In both cell-types, alkaloid biosynthesis related metabolites showed increases throughout the time-course, while in MCs phenylpropanoids showed increased levels (Fig 6). Epicatechin and 4-methylgenistein showed significant increase and decrease, respectively, in GCs. In addition, catechin and coumaric acid showed significant increases in GCs throughout the time-course. In MCs except 2,3-dihydroxybenzoic acid that showed significant increases throughout the time-course, no other metabolites showed significant changes (Fig 5). Moreover, GCs at 5 mpi, 15 mpi, and 30 mpi showed decreases in aromatic amino acids (tyrosine, phenylalanine, and tyrosine). These treatment-correlated metabolites indicate that the HCO_3_^-^-induced metabolic reprogramming involved the phenylpropanoid pathway and its branches such as flavonoid biosynthetic pathway. In addition, a clear remodeling of the phytohormonal status in both GCs and MCs was evident in HCO_3_^-^ treated cell-type metabolome, indicating increases in the majority of phytohormones in MCs and decreases in auxins and cytokinins in GCs (Fig 5). Interestingly, JA first increased in GCs, and then decreased at the later time-points thus coinciding with the stomatal closure (Fig 1). In contrast, the JA levels remained unchanged in MCs. Similarly, SA, kinetins (zeatin, zeatin riboside, and kinetin) and auxins (IAA and IBA) decreased in GCs, but increased in MCs. Only gibberellin content increased in GCs, and remained unchanged in MCs (Fig 6). Furthermore, HCO_3_^-^ treatment caused in GCs significantly increased GSH and decreased GSSG, dehydroascorbic acid, cysteine, cysteine-S-sulfate, and N-formylmethioine. In contrast, MCs contained more oxidized metabolites largely due to their increased photosynthesis and light-induced oxidation in the presence of high CO_2_ (Fig 5).

## Discussion

### Metabolomic insights into differential HCO_3_^-^ perception and conversion by GCs and MCs

Carbonic anhydrase converts CO_2_ into HCO_3_^-^ for incorporation into cellular metabolism [44] and acts as an upstream regulator of CO_2_ controlled stomatal movement [45–47]. Estimates indicate that at least 250 μM HCO_3_^-^ is present in the cytosol of a leaf cell in ambient air [48], maintained by cytosolic CA activity. Thus, the HCO_3_^-^ concentration used for the treatment is in excess. Photosynthesis is generally enhanced by elevated CO_2_, and leading to high accumulation of photosynthates [17, 49]. Furthermore, GC photosynthesis is critical for stomatal turgor production, but it does not mediate CO_2_ or ABA-induced stomatal closure [50]. With single-cell type metabolomics, we showed differential accumulation levels of metabolites in MCs and GCs, highlighting the power of single-cell type metabolomics in improving the resolution of tissue level metabolic changes [6]. Clearly, MCs contribute to the bulk of leaf metabolism and biosynthesis, whereas GCs present their unique metabolic repertoire for stomatal responses to environmental cues that range from pathogen to CO_2_. In GCs, the elevated HCO_3_^-^, more so than elevated CO_2_, alters the intracellular free calcium ion [Ca^2+^] sensitivity of SLAC1, leading to reduced stomatal opening [51]. In most non-photosynthetic tissues and the photosynthetic tissues of C3 plants, phosphoenolpyruvate carboxylase (PEPCase) catalyzes the conversion of phosphoenolpyruvate (PEP) and HCO_3_^-^ to oxaloacetate, i.e., to replenish TCA cycle intermediates in addition to malate production in guard cells [52]. Maintaining the cellular C/N balance in plants is complex and multiple mechanisms are involved [53, 54] to accommodate metabolome-wide changes.

In GCs, more metabolites showed HCO_3_^-^ responsiveness than in MCs as the capacity for metabolite flux through the catabolic pathways is high in GCs compared to MCs [55]. In addition, stomata and MCs play different roles in conducting CO_2_ to the sites of carboxylation in chloroplasts, as GCs are known to present higher PEPCase activities than MCs [56]. Thus, the large number of metabolites showing HCO_3_^-^ responsiveness in GCs may be due to high metabolic rates in different pathways. In fact, MCs are known to be involved primarily in photosynthate production, while GCs are gate keepers for air and water exchange, in addition to forming the first line of defense against pathogens. Thus, stomatal closure in response to increased HCO_3_^-^ helps to protect MCs from possible overloading. Increased accumulation of amino acids, phenylpropanoids, and redox metabolites in MCs contrasts with decreased accumulation of amino acids and increased accumulation of sugars, pentose phosphate pathway, and purine metabolites in GCs, reflecting the inherent functional dichotomy (Figs 5 and 6). Glutamate occupies a central position in amino acid metabolism and plays a central signaling and metabolic role at the interface of the C and N assimilatory pathways [57]. The decrease of glutamate in GCs (Fig 3B) reiterates the subdued amino acid metabolism. On the other hand, the boost in overall nitrogen metabolites in MCs reflects the primary functional role of MCs in incorporation of excess CO_2_ into N-metabolism. In contrast, GCs prominently direct carbon into sugars and pentose phosphate metabolites for energy. Our results further reflect the mutual dependency of GCs on MCs for cellular maintenance and regulatory functions.

### HCO_3_^-^ triggered changes in purine and specialized metabolism in MCs and GCs

Nitrogenous bases in the form of purines and pyrimidines form an essential pool of nitrogen in plant cells [58, 59] as metabolic intermediates, and signaling molecules in guard cell functions [9]. For instance, cyclic adenosine diphosphoribose (cADPR), a purine derived from nicotinamide adenine dinucleotide (NAD), plays important roles in guard cell ABA signaling. Injection of cADPR into guard cells resulted in [Ca^2+^] increases and turgor reduction [60]. Another purine, cyclic guanosine monophosphate (cGMP), has been implicated in ABA-induced stomatal closure by acting downstream of H_2_O_2_ and NO in the ABA signaling pathway [61]. The nitrated form of cGMP (8-nitro-cGMP) is a positive regulator in promotion of stomatal closure [62]. Moreover, increased accumulation of adenosine, inosine, and guanosine-derived metabolites in GCs was opposite to their decreased pattern in MCs, indicating their differential responses to elevated CO_2_. Pyrimidine metabolism operates mostly in plastids to provide intermediates for lipid and carbohydrate synthesis [63]. Decreased pyrimidine levels in GCs at later time-points indicate their possible utilization for the biosynthesis of lipids and sugars.

Phenylpropanoids are known in the context of plant stress and defense responses as they demonstrate a significant time-dependent accumulation [64]. MC-specific increases of phenylpropanoids as compared to GCs indicate that MCs may be the biosynthetic hubs for plant foliar defense systems as a result of phenylpropanoid accumulation [65, 66]. Transcripts involved in stress-responsive functions are known to be highly induced upon elevated CO_2_ treatment [43]. Flavonoids biosynthesis is downstream of phenylpropanoid metabolism. Although more prominent in GCs, comparable increases of flavonoids in both cell-types were noted (Figs 2 and 5). The presence of dihydroxy B-ring substituted flavonoids in the nucleus of MCs was reported [67], but their role other than protection from oxidative damage is unknown. Although the involvement of flavonoids in stomatal movement is known [9, 68], changes in flavonoid metabolism in response to elevated CO_2_ at the cellular levels have not been reported [69]. Evidently, the role of flavonoids such as kaempferol and quercetin in GCs is to protect from ROS induced stomatal closure [68]. Furthermore, in bread wheat [3] and strawberry [69] the accumulation of flavonoids increased upon elevated CO_2_ as compared to plants under ambient CO_2_. Similarly, elevated CO_2_ resulted in increased alkaloids by altering the C/N balance toward a positive effect on biosynthesis of these metabolites [70]. Under elevated CO_2_, high levels of morphine, codeine, papaverine, and noscapine were produced in wild poppy (*Papaver setigerum*) [71], and scopolamine was accumulated in jimson weed (*Datura stramonium* L.) [72]. In this study, we observed increased alkaloid biosynthetic pathway intermediates in both GCs and MCs. The increased specialized metabolites in both cell-types upon CO_2_ elevation may indicate increased primary metabolites/photosynthates, and signaling roles, e.g., in GCs.

### Redox state and phytohormone changes in HCO_3_^-^ treated MCs and GCs

NO is known to be a signaling intermediate in 2 mM HCO_3_^-^ induced stomatal closure in *Pisum sativum* [27], while ascorbate is known to be dramatically decreased by high CO_2_ in plants [19]. In this study, we showed that 1 mM HCO_3_^-^ induced stomatal closure (Fig 1). Although less severe in C3 plants, increased oxidative stress was found in plants grown under elevated CO_2_ [73, 74]. In addition, up-regulation of genes encoding enzymes involved in oxidative signaling and ROS scavenging such as glutathione-S-transferase, peroxidases, catalases, cysteine and thioredoxin-pathway related genes was reported [43]. Elevated CO_2_ can enhance maintenance of the redox potential due to an elevated rate of CO_2_ assimilation and low photorespiration [75]. Through H_2_O_2_ production and pyridine nucleotide interactions, photorespiration makes a key contribution to cellular redox homeostasis [76, 77]. Increased redox metabolites (e.g., homocysteine, methionine, methionine sulfone, N-formylmethionine, dehydroascorbate, glucoronate, gamma-glu-cys, and cystathionine) in MCs and their decreased and opposite accumulation patterns in GCs corroborate the role of redox state in GC signaling and stomatal movement [78]. Furthermore, the role of sulfur metabolites in GC signaling and movement has generated considerable interest. For instance, the role of sulfur dioxide (SO_2_) in NO and ROS-mediated apoptosis induction in the GCs of different species has been established [79–81]. Although changes of major phytohormones in response to stomatal ABA signaling were studied using metabolomic approaches [8], elevated CO_2_ is known to enhance the activities of ABA-independent enzyme, carbonic anhydrase, leading to further decrease in stomatal aperture [82]. Elevated CO_2_ is known to stimulate the SA pathway but repress the JA pathway in plants [83], as elevated CO_2_ may cause plants to re-allocate resources for SA synthesis and SA/JA crosstalk [84, 85]. Plant hormone signaling pathways related to defense are interconnected through complex regulatory networks [86]. In recently available transcriptomic data of moss gametophytes exposed to elevated CO_2_, up-regulation of ABA, JA, brassinosteroid, and ethylene metabolism-related genes was observed [43]. Similar instances were reported in *A*. *thaliana* challenged with elevated CO_2_ [14]. Not surprisingly, as foliar photosynthetic cells (i.e., MCs) form the bulk tissue in plants, the averaged leaf metabolic data share similarities to MC responses [14, 43]. Analysis of physiological and morphological stomatal responses of several species suggests that patterns of stomatal responses to CO_2_ do not follow a phylogenetic pattern associated with plant evolution [87]. This interesting phenomenon deserves further investigation using the specific cell-types and metabolomics approaches reported in this study.

## Conclusions

As time-resolved single-cell type metabolomic studies are rare, the HCO_3_^-^ induced metabolomic changes reported here in the two important cell-types help us understand high CO_2_-mediated short-term metabolomic responses in GCs and MCs. The responsiveness of the cells to HCO_3_^-^ is characterized by specific metabolites grouped by functional and temporal behavior. In MCs the increased metabolites outnumber the increased metabolites in GCs, where a comparable number of metabolites showed decreasing patterns. Moreover, the metabolomic responses of MCs showed contrasting patterns to GCs in amino acid, purine, phytohormone, and flavonoid metabolism in response to elevated HCO_3_^-^, which reflects the differential metabolic predisposition and responses to the stimulus. Hence, the results in this study have shown utility of metabolomics tools towards improved understanding of the plant CO_2_ sensing and response mechanisms. The data may be useful to predict plant responses and productivity in an ever changing climate. In addition, efforts are underway to enhance C3 carbon concentrating mechanism in chloroplasts by adding more bicarbonate transporters to increase bicarbonate flux into plastids for enhanced carbon fixation [88–90]. We are fully aware that GCs are not autonomously regulated, and there is a strong link between MC metabolism and stomata function [91, 92]. Focused studies on the interactions between MCs and GCs are underway in our laboratories.

## Supporting Information

Additional Supporting Information may be found in the online version of this article at the publisher’s web-site:

S1 Fig Total metabolites quantified mapped onto KEGG metabolic pathways. Using targeted HPLC-MRM-MS and GC-MS platforms, a total of 268 metabolites (shown as red dots) were quantified and mapped onto KEGG pathways.
(TIF)

S2 Fig Volcano plots displaying differential changes of metabolite levels in MCs at different time-points of HCO_3_^-^ treatment compared to respective controls. (A) 5 mpi, (B) 15 mpi, (C) 30 mpi, (D) 60 mpi, and (E) 120 mpi HCO_3_^-^ treatment. Metabolites are ranked according to their statistical–log10 (P-value) (y-axis) and log 2 (fold change) (x-axis). Cut-offs were P-values < 0.05 and fold changes > 1.2 or < 0.8. Off-centered metabolites are those that varied the most between the two treatment conditions.
(TIF)

S3 Fig Volcano plots displaying differential changes of metabolite levels in GCs at different time-points of HCO_3_^-^ treatment compared to respective controls. (A) 5 mpi, (B) 15 mpi, (C) 30 mpi, (D) 60 mpi, and (E) 120 mpi HCO_3_^-^. Metabolites are ranked according to their statistical–log10 (P-value) (y-axis) and log 2 (fold change) (x-axis). Cut-offs were P-values < 0.05 and fold changes > 1.2 or < 0.8. Off-centered metabolites are those that varied the most between the two treatment conditions.
(TIF)

S4 Fig Uniquely identified metabolites in the two cell-types. (A) MCs and (B) GCs. Abbreviations used: ME: methyl ester, 2H4MAP: 2-hydroxy4-methoxyacetophenone.
(TIF)

S5 Fig Total increased, decreased, and significantly changed metabolites during the time-course profiling study. (A) MCs and (B) GCs. Significantly changed metabolites include both increased and decreased metabolites at a given time-point.
(TIF)

S1 Table Metabolites used in the time-course profiling study for the targeted analysis by HPLC-MRM-MS/MS. KEGG: Kyoto Encyclopedia of Genes and Genomes, HMDB: Human Metabolome Database, InChI Key: IUPAC International Chemical Identifier, CAS: Chemical Abstracts Service (CAS) number, ESI: Electron Spray Ionization, Q1:Precursor Ion, Q2: Daughter Ion (Transition), DP: Declustering Potential, CE: Collision Energy, CXP: Cell Exit Potential (10 and -10 for positive and negative modes for the 4000 QTRAP (ABSciex) used.
(XLSX)

S2 Table Raw metabolomic data sets. Peak areas (as relative abundances) of metabolites quantified using combined GC-MS and HPLC-MRM-MS/MS analyses at 0, 5, 15, 30, 60, and 120 mpi bicarbonate treatment (T) and control (C) for GCs and MCs (n = 4).
(XLSX)

S3 Table Processed metabolomic data sets for MCs. Fold changes (1.2 and 0.8 as cut-offs) and significantly changed MC metabolites (P < 0.05) at 5, 15, 30, 60 and 120 mpi bicarbonate treatment with respect to controls as inferred from ANOVA.
(XLSX)

S4 Table Processed metabolomic data sets for GCs. Fold changes (1.2 and 0.8 as cut-offs) and significantly changed GC metabolites (P < 0.05) at 5, 15, 30, 60, and 120 mpi bicarbonate treatment (T) with respect to controls (C) as inferred from ANOVA.
(XLSX)

S5 Table Significantly changed metabolites in the cell-types as inferred by within subject ANOVA. Significantly changed metabolites (P < 0.05) for (A) GC and (B) MC at 5, 15, 30, 60 and 120 mpi bicarbonate treatment with respect to controls as inferred from within subject ANOVA models.
(XLSX)

S6 Table Significantly changed metabolites in two cell-types. Significantly changed metabolites (P-value< 0.05) for (A) GC and (B) MC at 5, 15, 30, 60 and 120 mpi bicarbonate treatments with respect to controls.
(XLSX)

S7 Table Pathway enrichment of changed metabolites in the two cell-types. Pathway enrichment for (A) GC and (B) MC metabolites showing increases or decreases after 5, 15, 30, 60, and 120 mpi bicarbonate treatment.
(XLSX)

## Abbreviations

GCs: guard cells
MCs: mesophyll cells
HCO_3_^-^: bicarbonate
mpi: minutes post-incubation
PEPCase: phosphoenolpyruvate carboxylase
MRM: multiple reaction monitoring
HPLC: high performance liquid chromatography
GC: gas chromatography
LC: liquid chromatography
MS: mass spectrometry
