# Supplementary figures and images for: Metabolomic Responses of Guard Cells and Mesophyll Cells to Bicarbonate
Source: PLoS One. 2015 Dec 7;10(12):e0144206. doi: 10.1371/journal.pone.0144206 (PMC4671721; doi:10.1371/journal.pone.0144206)

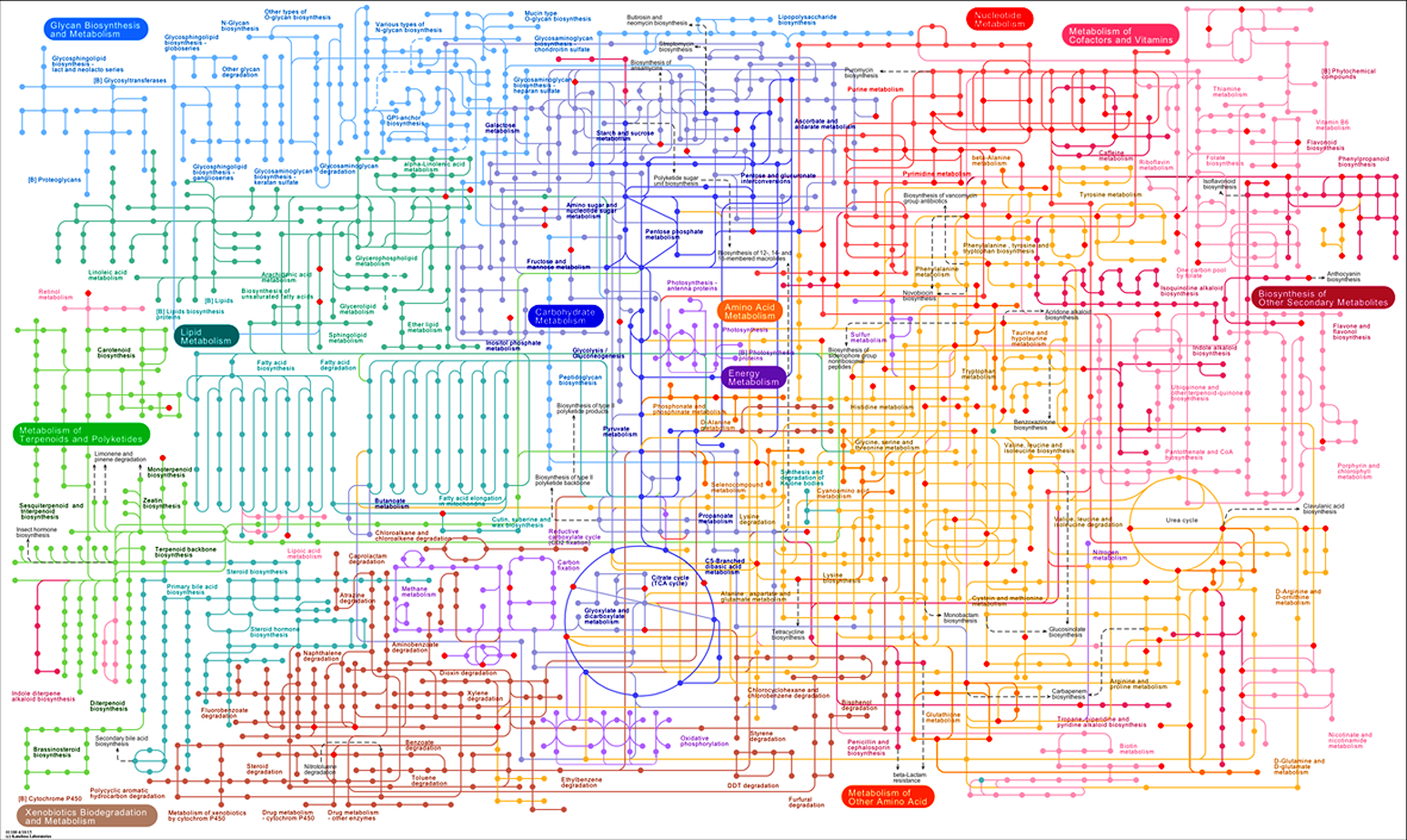

Supplement: S1 Fig — Using targeted HPLC-MRM-MS and GC-MS platforms, a total of 268 metabolites (shown as red dots) were quantified and mapped onto KEGG pathways. (TIF) [file pone.0144206.s001.tif]

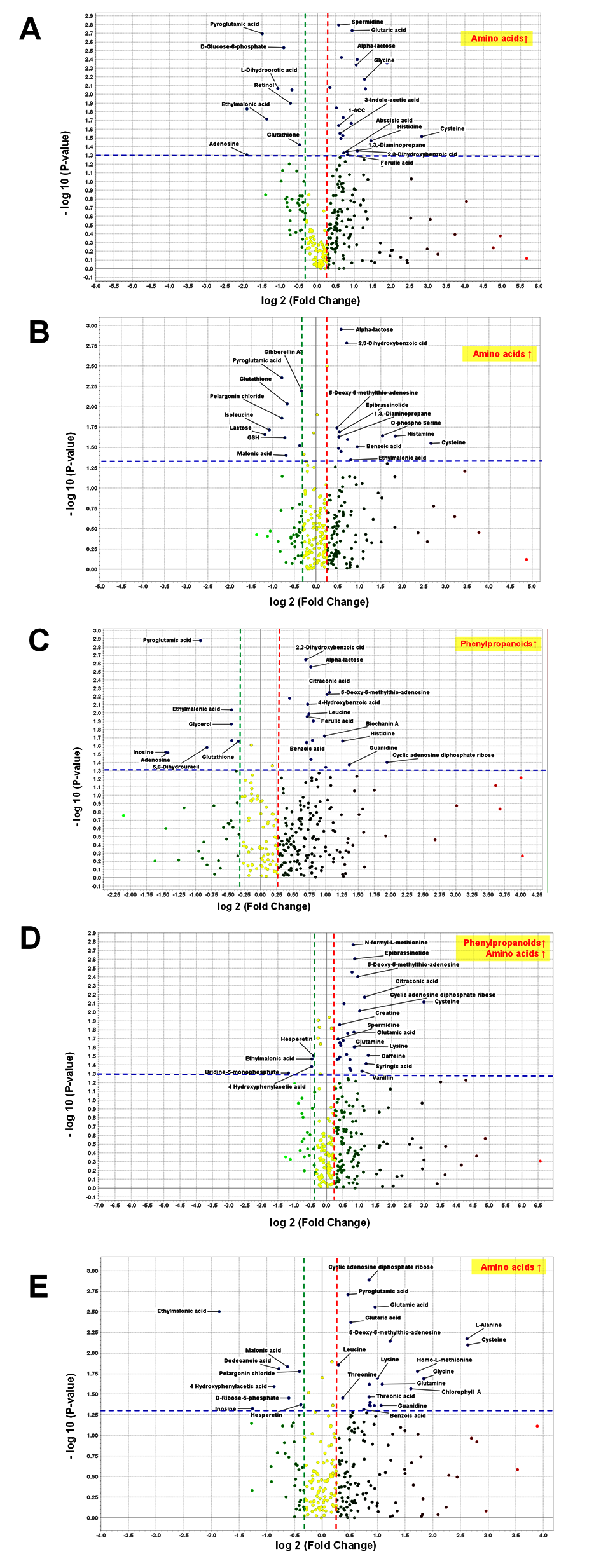

Supplement: S2 Fig — (A) 5 mpi, (B) 15 mpi, (C) 30 mpi, (D) 60 mpi, and (E) 120 mpi HCO3 - treatment. Metabolites are ranked according to their statistical–log10 (P-value) (y-axis) and log 2 (fold change) (x-axis). Cut-offs were P-values < 0.05 and fold changes > 1.2 or < 0.8. Off-centered metabolites are those that varied the most between the two treatment conditions. (TIF) [file pone.0144206.s002.tif]

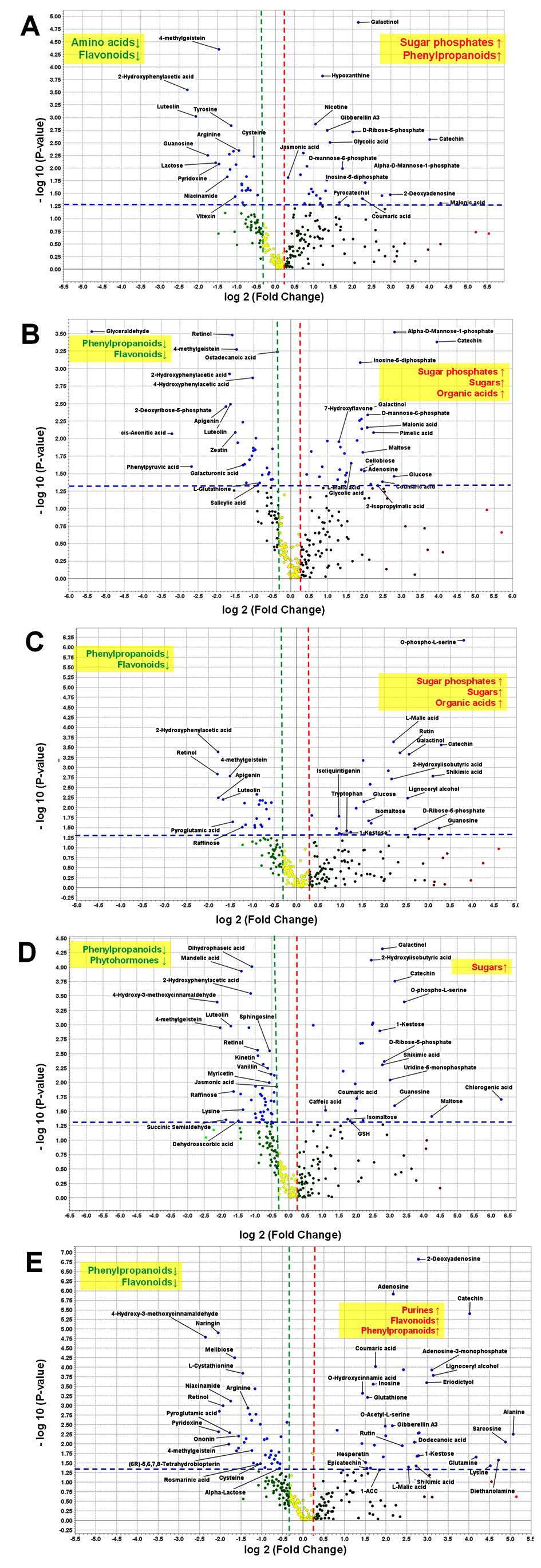

Supplement: S3 Fig — (A) 5 mpi, (B) 15 mpi, (C) 30 mpi, (D) 60 mpi, and (E) 120 mpi HCO3 -. Metabolites are ranked according to their statistical–log10 (P-value) (y-axis) and log 2 (fold change) (x-axis). Cut-offs were P-values < 0.05 and fold changes > 1.2 or < 0.8. Off-centered metabolites are those that varied the most between the two treatment conditions. (TIF) [file pone.0144206.s003.tif]

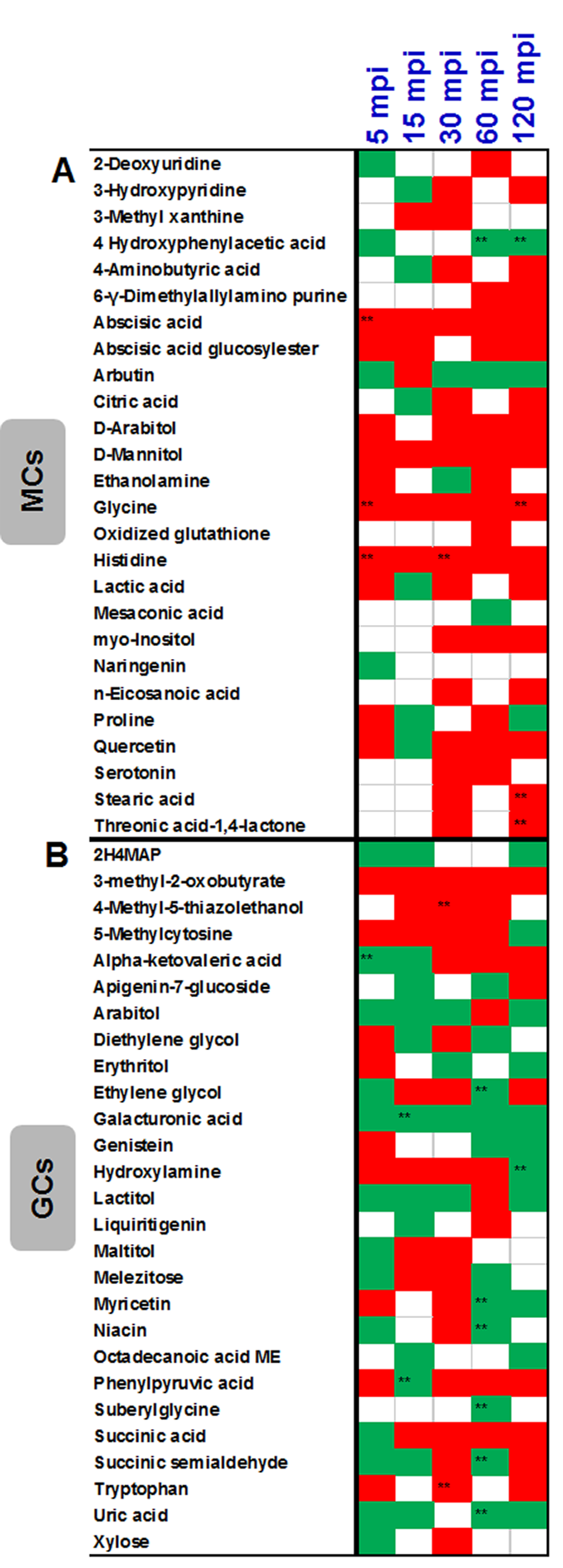

Supplement: S4 Fig — (A) MCs and (B) GCs. Abbreviations used: ME: methyl ester, 2H4MAP: 2-hydroxy4-methoxyacetophenone. (TIF) [file pone.0144206.s004.tif]

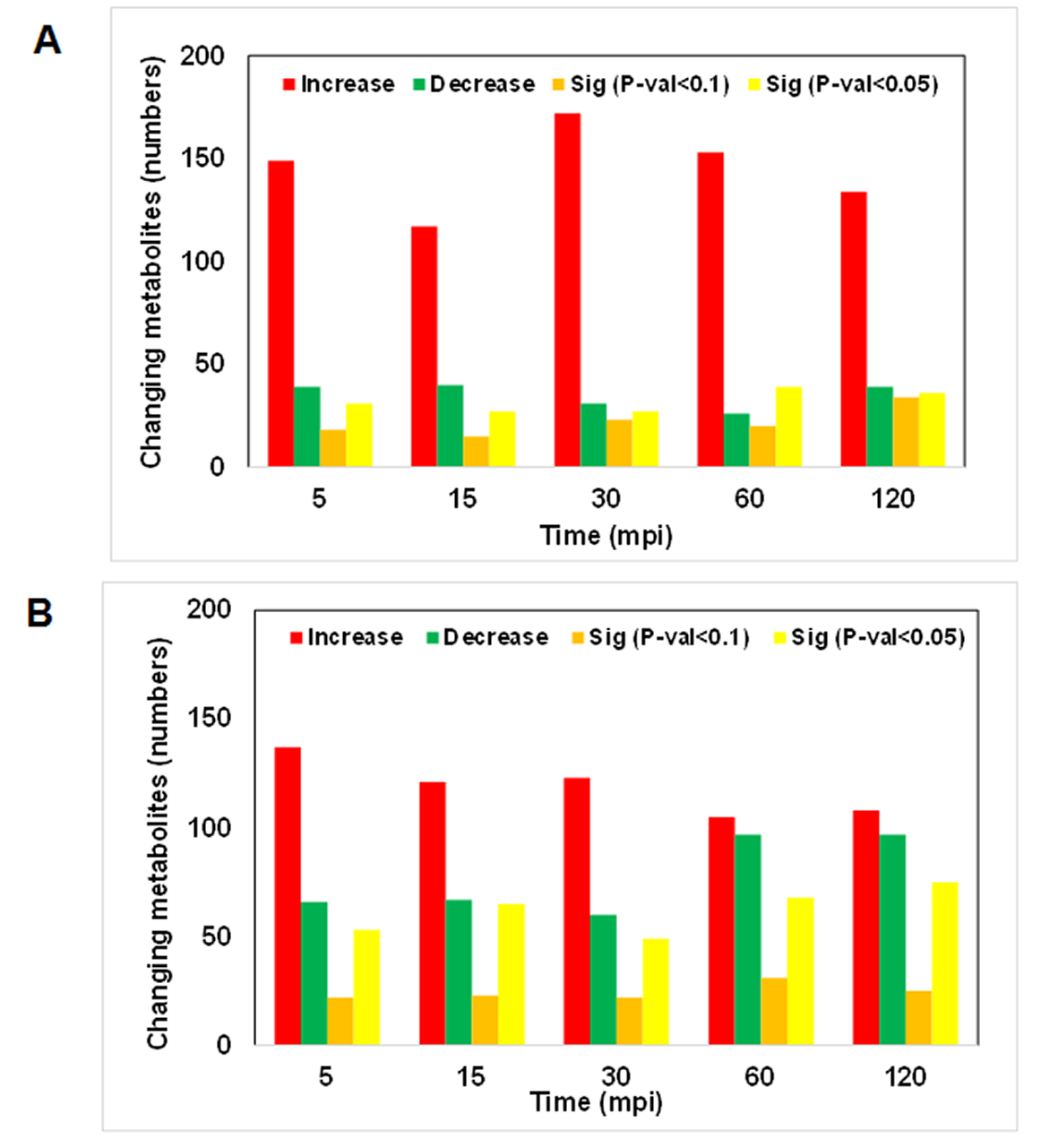

Supplement: S5 Fig — (A) MCs and (B) GCs. Significantly changed metabolites include both increased and decreased metabolites at a given time-point. (TIF) [file pone.0144206.s005.tif]
